# Comparative fixation devices for preventing migration of the proximal tibiofibular joint during tibial lengthening: a tether versus screw fixation

**DOI:** 10.1186/s13018-023-03771-z

**Published:** 2023-07-15

**Authors:** Jidapa Wongcharoenwatana, Jason S. Hoellwarth, Michael D. Greenstein, Taylor J. Reif, Austin T. Fragomen, S. Robert Rozbruch

**Affiliations:** 1 Limb Lengthening and Complex Reconstruction Service, Hospital for Special Surgery, New York, NY USA; 2 Department of Orthopaedic Surgery, Faculty of Medicine, Siriraj Hospital, Mahidol University, Bangkok, Thailand

**Keywords:** Tibial lengthening, Internal lengthening nail, Distraction osteogenesis, Magnetic nail, Proximal tibiofibular joint, Fixation, Fibular migration

## Abstract

**Background:**

When lengthening the tibia segment using motorized internal lengthening nails (MILN), undesired distal migration of the proximal fibula segment is prevented by tibiofibular stabilization, traditionally using a screw. A tightened cortical suspensory fixation rope (tether) is an alternative option, but its appropriateness has never been studied. The primary outcome was comparing the amount of proximal fibular migration between patients who were stabilized with either a tether or a screw. The secondary outcome was to evaluate the effect of fibular migration on the clinical outcome between both groups.

**Methods:**

A retrospective study was conducted on patients who underwent tibial lengthening with MILN between April 2016 and June 2022. Two cohorts were compared: 18 limbs with tether fixation versus 29 limbs with screw fixation. Data on the patient's age, sex, etiologies, and clinical outcomes were collected. Radiographic measurements included the lengthening distance and the amount of proximal fibular migration.

**Results:**

In total, 47 limbs from 41 patients, with average age 35.01 ± 13.72 years old. There were 28 males (68.29%) and 13 females (31.71%). The tether group demonstrated a statistically significant greater distance of migration than the screw group (*p* < 0.001), with an average migration distance of 8.39 ± 5.09 mm and 2.59 ± 3.06 mm, respectively. No correlation was found between the amount of tibial lengthening and the distance of proximal fibular migration in both the tether group (*p* = 0.96) and the screw group (*p* = 0.32). There was no significant difference in the change of knee extension between both groups (*p* = 0.3), and no patients reported knee pain or tightness.

**Conclusion:**

A screw provides better resistance to proximal tibiofibular joint migration during MILN lengthening, but the difference appears clinically inconsequential. Either option appears suitable.

## Introduction

Tibial lengthening can be performed using a motorized internal lengthening nail (MILN) for many indications, such as to address limb length discrepancy arising from congenital, traumatic, developmental, or other etiologies [1, 2]. Stabilization of the fibula to the tibia is recommended during tibial lengthening in order to prevent distal migration of the proximal fibula segment, leading to deformity and contracture of the knee [2]. Conventionally, screws have been used for surgical stabilization of the proximal tibiofibular joint due to their strength and stability [3]. However, achieving good bone purchase in both the tibia and fibula while avoiding the nail can sometimes be challenging. An alternative fixation option is a tightened cortical suspensory fixation rope (tether) [1, 4], which has proven successful in providing physiologically suitable fixation of other joints, most notably the distal tibiofibular joint [5, 6]. However, the tether’s suitability in proximal tibiofibular fixation during tibial MILN lengthening has never been evaluated.

To address this knowledge gap, for surgical benefit, the current study compares the use of a tether or a screw for proximal tibiofibular joint fixation during tibial lengthening with MILN. Using the radiographic measurement of proximal tibiofibular joint (fibular head) migration to determine the difference in the fixation stability. The primary outcome was the amount of radiographic proximal tibiofibular joint migration. The secondary outcome was the clinical impact of fibular migration, specifically knee motion, pain, and peroneal nerve deficit.

## Methods

Following Institutional Review Board approval, we retrospectively reviewed our database of tibial MILN lengthening performed between April 2016 and June 2022.

### Patient selection

Two cohorts were created to perform a comparison: one of patients with tether fixation and the second with screw fixation. Tether fixation has been the preferred option of one of the authors starting in 2020. Inclusion criteria were all patients with tether fixation who had complete anterior–posterior tibia radiographs performed preoperatively and at lengthening completion: this identified 18 limb segments in 21 patients. A comparison cohort was compiled by identifying 29 tibial MILN procedures performed by this author and two other co-authors during the same and immediately preceding 2 years, also with a complete radiographic series. Patients were excluded if they: had incomplete radiographic or clinical records or were still lengthening at the time of this study. Forty-one patients with a total of 47 limbs were enrolled, and data on patients’ age, sex, etiologies, and clinical outcomes were collected.

### Surgical technique

All patients had tibial and fibular osteoplasty performed by one of three fellowship trained limb reconstruction surgeons based on published techniques [1]. After the nail was inserted and fixed with locking screws, stabilization of the proximal tibiofibular joint was achieved with either a tether (TightRope® System, Arthrex, Naples, FL, USA) or with a 5-mm fully threaded screw inserted from the fibula into the tibia.

### Lengthening protocol and follow-up

Lengthening typically commenced on the seventh postoperative day with rate and rhythm dictated by patient factors, but a maximum of 0.2 mm 4 times per day until the desired length was obtained. Patients were evaluated clinically and radiographically every 2–3 weeks until the end of the lengthening goal was achieved; lengthening rate and rhythm was adjusted based on radiographic and/or clinical assessment. Full weight-bearing was allowed when bridging bone was seen at 2–3 cortices of the regeneration.

At preoperative and each postoperative visit, patients were asked whether they experienced pain in the knee. The final knee pain evaluation was done at the time of nail removal, approximately 1 year postoperative. Physical examination before and at each visit included prone knee flexion and extension, supine ankle flexion and extension, and patellar tracking. We also evaluated peroneal nerve function.

### Data collection and statistical analysis

**Fig. 1:**
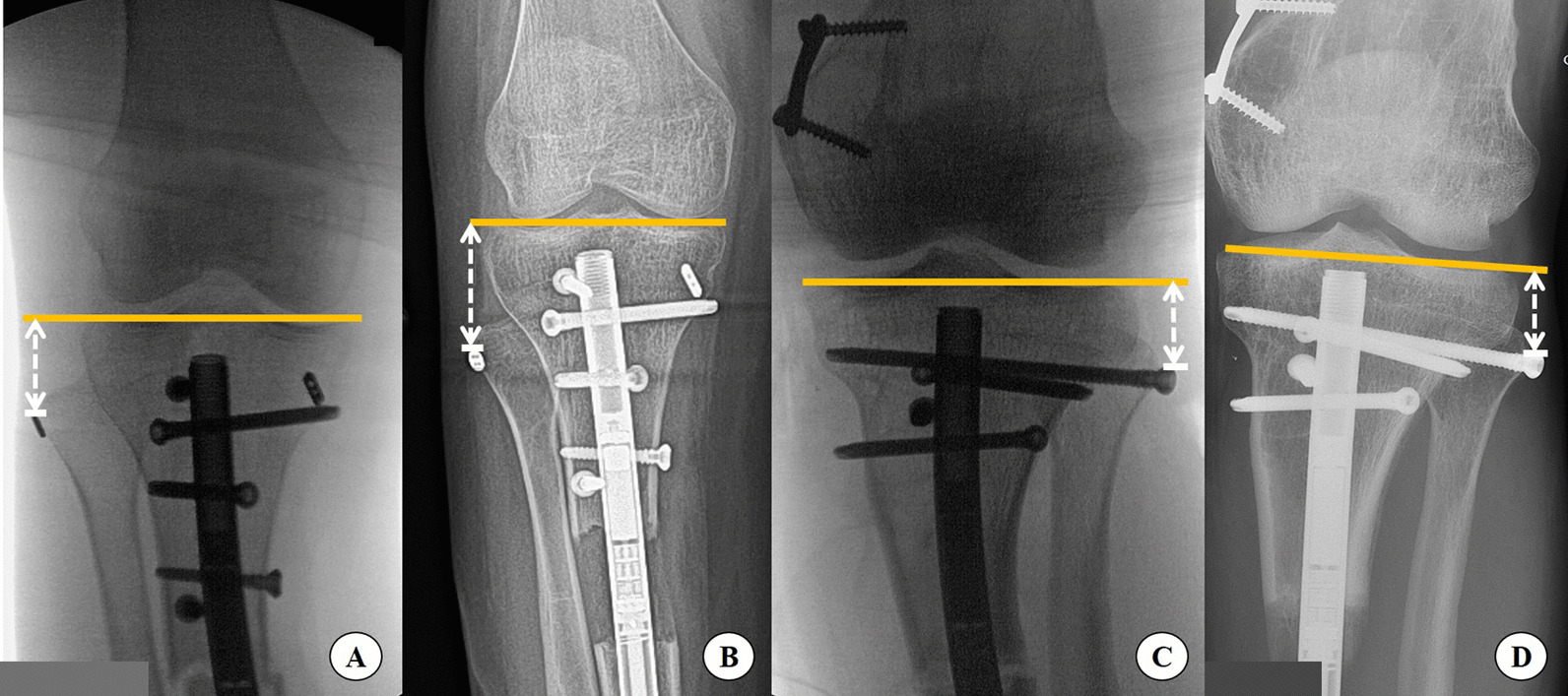
Radiographic measurements of the difference of proximal fibular migration distance from sample patients. In the tether group, the immediate postoperative distance was 26 mm (**A**) and 37 mm at the end of lengthening (**B**). In the screw group, the immediate postoperative distance was 22 mm (**C**) and 25 mm at the end of lengthening (**D**)

**Fig. 2:**
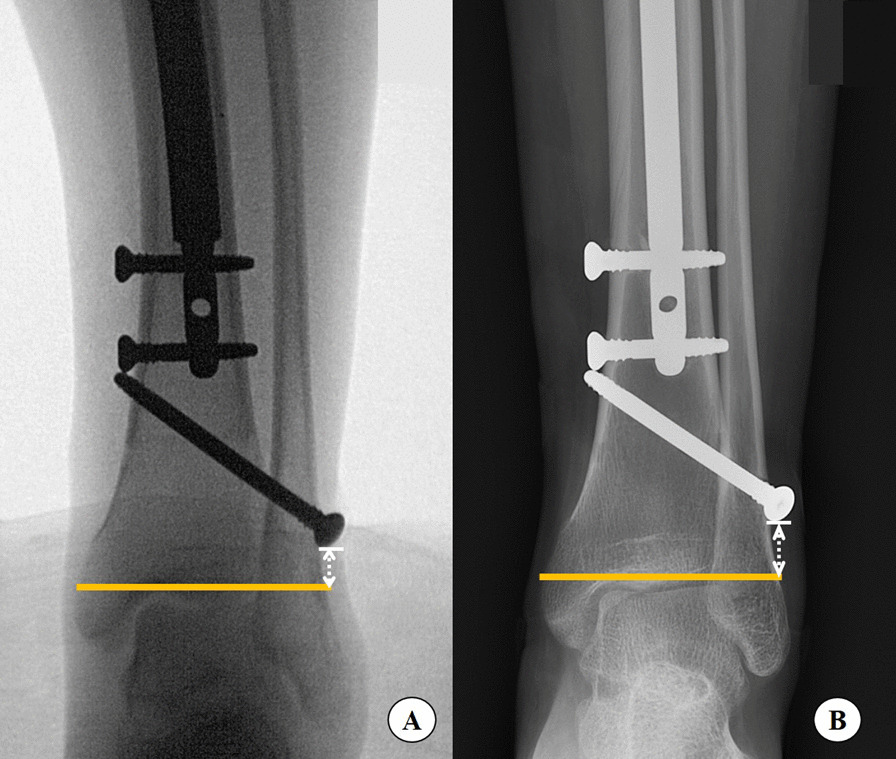
Radiographic measurements of the difference of distal fibular migration distance from sample patients. The immediate postoperative distance was 8 mm (**A**) and 11 mm at the end of lengthening (**B**)

**Fig. 3:**
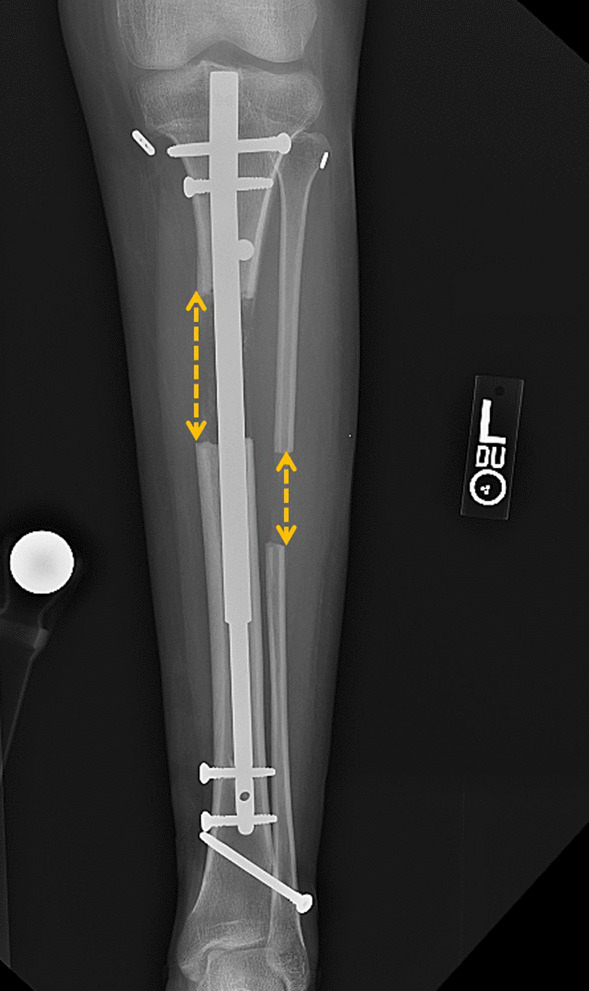
Radiographic measurements at the end of lengthening showed tibial was lengthened 50 mm and fibular was lengthened 32 mm. Fibular/tibial lengthening ratio was 64%

Radiographic evaluation was performed at specific phases. The baseline proximal and distal tibiofibular position was established using immediate postoperative tibia radiographs. During lengthening, dedicated tibia radiographs were taken with the patient not weight-bearing. The total proximal fibular migration distance was determined by the distance between the tip of tether or screw relative to the line drawn between the medial and lateral tibial plateau (Fig. 1), the distal fibular migration distance was measured as the distance between the tip of screw relative to the line drawn parallel to ankle joint line (Fig. 2). The difference of the distance in both parameters were evaluated at the end of lengthening versus the immediate postoperative radiograph, altogether with tibial and fibular lengthening distance (Fig. 3). The migration ratio was calculated by dividing migration distance by the total lengthening achieved.

Comparisons between groups including patients’ demographic data, lengthening distance, magnitude, and direction of migration were calculated. The Kolmogroff-Smirnov normality test was performed for each data category; normally distributed data were compared using Student’s t-test; non-normally distributed data were compared using the Mann–Whitney U test. The relationship between the magnitude of lengthening with the direction and magnitude of tibiofibular migration was assessed using Pearson correlation. Significance was set as *p* < 0.05. Statistical calculation was performed using DATAtab: Online Statistics Calculator. DATAtab e.U. Graz, Austria, E.U. https://datatab.net.

## Results

### Demographic study

In total, 47 limbs were evaluated from 41 patients, with the average age being 35.01 ± 13.72 years old. There were 28 males (68.29%) and 13 females (31.71%). Indications for lengthening were to address post-traumatic (23.4%), congenital (42.55%), endocrine and metabolic (6.38%), and other (27.66%) etiologies. There were 18 limbs in the tether group (38.3%), and 29 limbs (61.7%) in the screw fixation group. Table 1 summarizes each cohorts’ demographics.

**Table 1:** Demographic data

	Tether (*n* = 18)	Screw (*n* = 29)	*p* value
Age (years)	31.69 ± 13.21 (15–61)	35.72 ± 14.3 (14–63)	0.42^†^
Sex [*n*, (%)]			
Male	11(61.11)	21(72.41)	0.2
Female	7(38.89)	8(27.59)	
Laterality [*n*, (%)]			
Left	7(38.89)	12(41.38)	0.87
Right	11(61.11)	17(58.62)	
Indication for surgery [*n*, (%)]			
Post-traumatic	2(4.26)	9(19.15)	0.09
Congenital	11(23.4)	9(19.15)	
Endocrine and metabolic causes	2(4.26)	1(2.13)	
Others	3(6.38)	10(21.28)	
Follow-up (months)	11.33 ± 6.35 (2.83—27.03)	25.58 ± 15.78 (2.13—68.00)	< 0.001*^†^

Data in columns are presented as Mean ± SD, (range), * = *p* < 0.05 was considered significant. Comparisons were performed using Student’s *t*-test, except for non-normal distributions which were compared using the Mann–Whitney U test (†)

### Radiographic evaluation

**Fig. 4:**
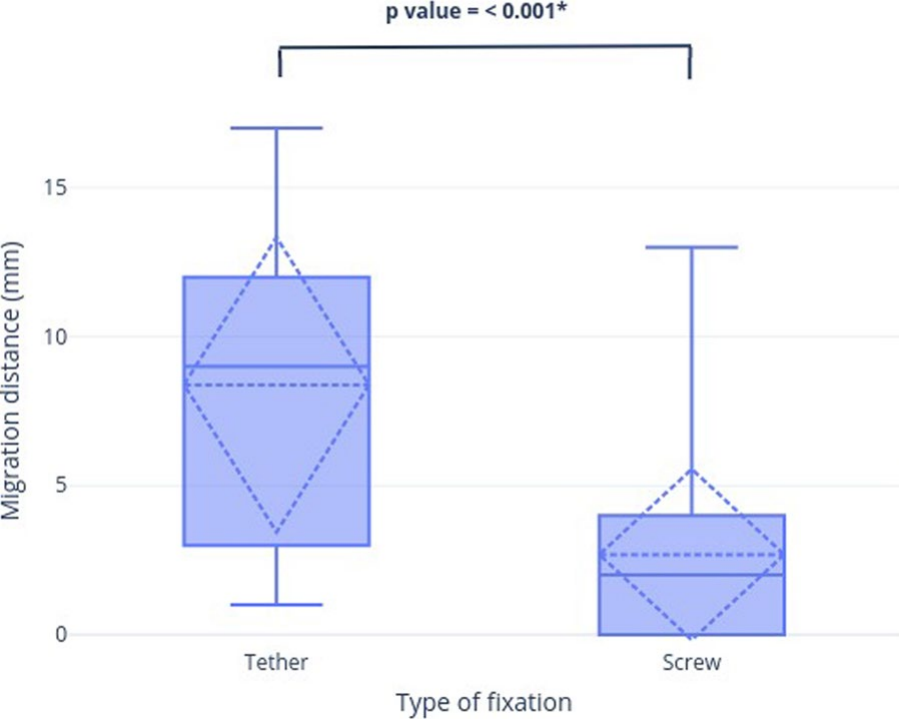
Box plot of proximal tibiofibular migration distance between tether and screw fixation groups with Mean and SD

**Fig. 5:**
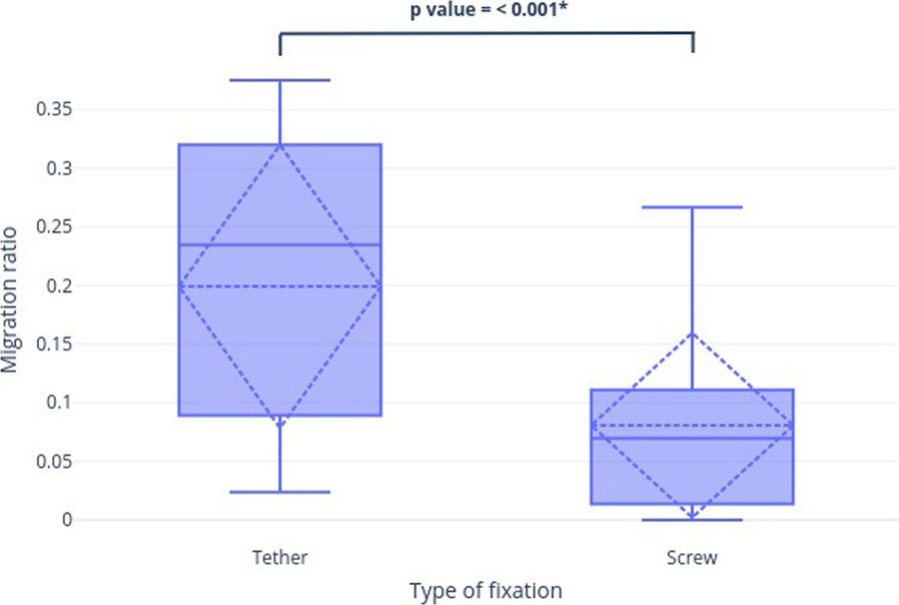
Box plot of proximal tibiofibular migration ratio between tether and screw fixation groups with Mean and SD

The primary outcome measure of this study was the proximal tibiofibular joint migration as measured radiographically. The data were summarized in Table 2 and shown graphically in Figs. 4 and 5. The tether group had significantly greater distal migration than the screw group (8.39 ± 5.09 vs. 2.59 ± 3.06 mm, *p* < 0.001). Pearson analysis identified a very poor correlation between tibial lengthening achieved and proximal tibiofibular joint migration distance in both the tether (*r* = − 0.01, *p* = 0.96) and the screw (*r* = 0.19, *p* = 0.32) groups. Of 47 limbs, only 27 limbs had distal tibiofibular joint screw fixation. The remaining limbs were not fixed for various reasons such as patients who underwent post-ankle fusion or distal fibular bone resected. Distal tibiofibular migration distances in both tether and screw groups were not significantly different, with averages of 1.8 ± 1.9 mm and 2.33 ± 1.37 mm, respectively (Table 3). Additionally, there was no evidence of valgus deformities or premature consolidation of the fibula in any patients from both groups.

**Table 2:** Comparison of radiographic and clinical outcomes between tether and screw fixation

	Tether (n = 18)	Screw (n = 29)	*p* value
*Radiographic parameters*			
Tibial lengthening achieved (mm)	44.94 ± 11.49 (18–65)	37.76 ± 12.37 (16–56)	0.05^§^
Fibular lengthening (mm)	31.11 ± 14.66 (5–54)	24 ± 15.82 (3–50)	0.61^§^
Fibular/Tibial lengthening ratio (%)	66.53 ± 20.86 (14.29–96.15)	62.34 ± 29.85 (6–94.12)	0.13^§^
Proximal tibiofibular migration distance (mm)	8.39 ± 5.09 (1–17)	2.59 ± 3.06 (0—13)	< 0.001*^†^
Proximal tibiofibular migration ratio	0.20 ± 0.07 (0.02–0.4)	0.07 ± 0.09 (0.1–0.3)	< 0.001*^†^
*Clinical outcomes*			
Change in knee extension (degrees)	1.83 ± 4.19 (0–15)	1.66 ± 3.30 (0–10)	0.3^†^
Change in knee flexion (degrees)	7.50 ± 10.33 (0–35)	8.10 ± 11.05 (0–40)	0.8^†^

Data in columns are presented as Mean ± SD, (range), * = *p* < 0.05 was considered significant,

^§^Comparisons were performed using a *t*-test

^†^Comparisons were performed using the Mann–Whitney U test

**Table 3:** Comparison of distal tibiofibular joint migration between tether and screw fixation

	Tether (*n* = 15)	Screw (*n* = 12)	*p* value
*Radiographic parameters*			
Distal tibiofibular migration distance (mm)	1.8 ± 1.9 (0–6)	2.33 ± 1.37 (0–4)	0.41^§^
Distal tibiofibular migration ratio	0.05 ± 0.06 (0–0.17)	0.06 ± 0.05 (0–0.2)	0.43^§^

Data in columns are presented as Mean ± SD, (range), * = *p* < 0.05 was considered significant,

^§^Comparisons were performed using a *t*-test. The number of patients (15 and 12) is not the entire cohort because some patients had prior surgery which obviated the need for the fibular length stabilization screw

### Clinical evaluation

**Fig. 6:**
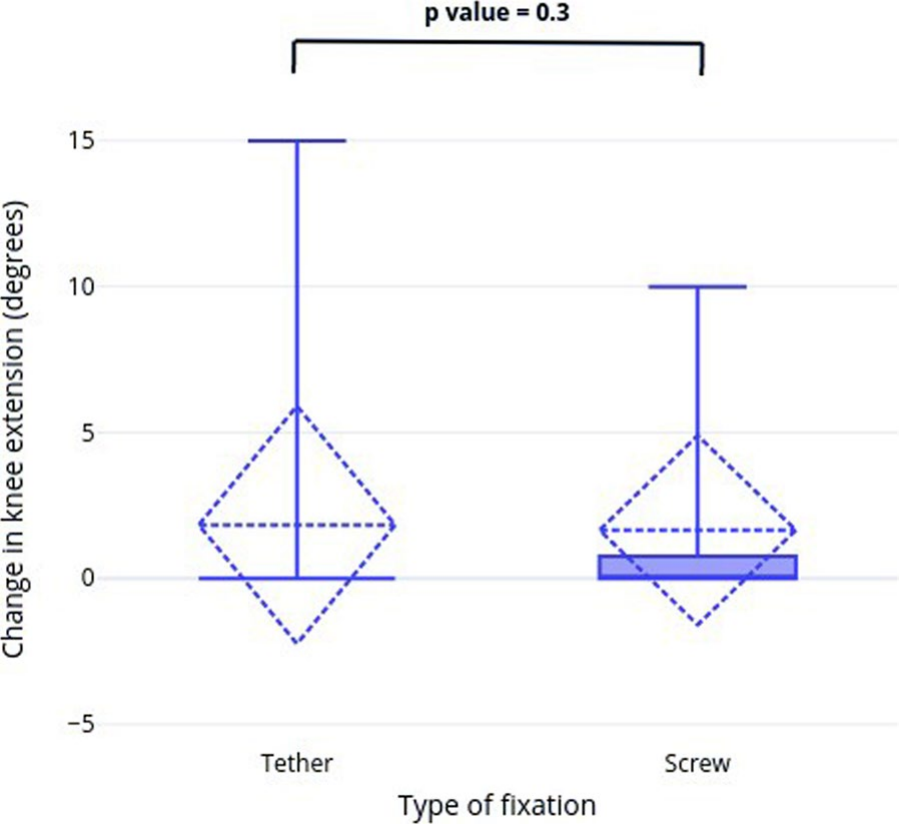
Box plot of change in knee extension after tibial lengthening between tether and screw fixation groups with Mean and SD

The secondary outcome measure was the clinical impact related to the proximal tibiofibular joint migration. All patients in both groups achieved their lengthening goal, with no clinical complaints at the final follow-up. Specifically, no patient expressed or had examination evidence of proximal tibiofibular joint area swelling, tightness, localized pain, or tenderness. No evidence of peroneal nerve sensory deficit or muscle weakness was found in any patient. The average change in knee extension between preoperative and final follow-up was not significantly different between both groups (*p* = 0.3) (Fig. 6).

## Discussion

The most important finding of this study was that the screw fixation provides significantly better stabilization measured by radiograph, but without any clinically recognizable difference related to pain, knee motion, or nerve function. There does not appear to be any relationship between the amount of lengthening to the amount of proximal tibiofibular migration. Although the amount of proximal fibular migration in tether patients was significantly greater than in the screw fixation group, there was no significant difference in any of the clinical measures: knee motion, pain, and peroneal nerve dysfunction.

This is the first study regarding the use of a tether fixation of the proximal tibiofibular joint during tibial MILN lengthening, thus the discussion will focus on related literature addressing similar clinical scenarios. Numerous studies demonstrated distal migration of the fibular head during lengthening [7–14], with greater distance migrated consistently observed when the tibiofibular joint had no fixation [10]. However, Song et al. [15] demonstrated that fibular migration could occur even if the joint is fixed with a tensioned wire. In 2003, Kashiwagi et al. compared various types of proximal tibiofibular joint fixation and concluded that a half pin provided the best fixation compared to a screw or a wire [16]. However, the half pin does not permit a major benefit of MILN: completely internal implants. Screws have been recommended to stabilize the proximal tibiofibular joint, and some studies suggested solid 4.5 mm screws rather than smaller size or cannulated screws, which were more likely to break [1, 7]. Consistent with prior literature, our study also demonstrated proximal fibular migration in both groups, with the amount of migration in the tether group being significantly greater than in the screw fixation group. But whereas Hatzokos [13] reported that the magnitude of fibula migration was linearly correlated with the amount of tibial lengthening, the correlation was not observed in the current study. We feel the tether reaches a sufficient tension to impede further migration during the initial lengthening period and thus additional lengthening does not lead to linear increases in migration. The screw functions similarly, but with greater stiffness and reaches sufficient force much faster.

Proximal fibula migration can be associated with clinically meaningful symptoms. Boero et al. demonstrated no association between proximal fibular migration up to 41 mm with worsening knee function or malalignment [10]. This may be due to the gradually elongated proximal tibiofibular joint capsule [13]. The results from our study identified that no patients experienced a clinically detectable symptom (specifically knee range of motion, pain, or peroneal nerve deficit), even with up to maximum migration of 17 mm. Even though patients with both types of fixations showed migration of the proximal fibula during tibial lengthening, there were no significant changes in knee extension. Knee extension can often become a problem during the lengthening experience (usually due to taut hamstrings), and some patients in our study also experienced temporary knee extension deficits, but almost all had completely resolved by the end of the follow-up period by improving adherence with physical therapy and/or adjusting the lengthening rate. In the distal tibiofibular joint, a previous study comparing screws and tether fixation demonstrated similar results on both ankle dorsiflexion and plantarflexion [17]. McCartan et al. reported tether fixation showed no restricted ankle dorsiflexion and the mean total ankle range of motion was comparable to the other side [18].

Beyond clinical symptoms, the main risk related to fibular head migration is premature consolidation of the fibular osteotomy site [15]. Kim et al. showed that proximal fibular migration of more than 10 mm was associated with premature consolidation which induced knee valgus deformity [9]. In our study, there were some patients with up to 17 mm of proximal fibular migration but no premature fibular consolidation or valgus deformities occurred. Literature regarding tethered fixation of the proximal tibiofibular joint, for use in non-MILN situations, identifies the primary associated risk is persistent peroneal nerve symptoms [19]. No patients in this study experienced any peroneal nerve symptoms.

Consistent with the above studies, we believe proximal tibiofibular fixation is necessary to prevent large fibular migrations and subsequent premature consolidation/valgus deformity. The tether fixation has been used widely for joint stabilization, though not in the setting of distraction. So the following considerations led us to consider the use of a tether for fixation versus the typical screw [1, 7]. The screw head prominence at the fibular head irritates many patients for the entirety of the time before screw removal, usually a year after the index surgery. The tether button has a low enough profile that it rarely causes patient discomfort and our experience was consistent with this benefit [6, 20–22]. In response to the forces of lengthening, some screws can break [23], and certainly, a tether can loosen or break as well. However, a symptomatic broken screw is more difficult to fully remove and the residual metal in the proximal tibia is more likely to cause future problems. Other authors have left tethers long-term and we have done that as well. This would also affect the overall cost of each implant. The cost of screws is around 300 USD, whereas the tether is approximately 600–1000 USD. However, the tether is considered more cost-effective with screw removal surgery (routinely or due to a symptomatic broken screw). [24, 25] According to our study, using tethers for proximal tibiofibular joint fixation during tibial lengthening with MILN demonstrated sufficient stabilization to achieve equal clinical outcomes versus screw fixation.

Limitations necessary to consider include the small sample size of each compared group, the potential for selection bias due to the retrospective design of the study, and the heterogeneity of patient age, sex, size, etiologies for lengthening, and magnitude lengthened. Strengths include the full availability of clinical and radiographic records for all patients and the consistency of technique among the surgeons. Importantly, as there is no previous study on this specific topic, an additional strength is the primacy of providing information on the topic.

## Conclusion

Screw fixation of the proximal tibiofibular joint during MILN lengthening provides better resistance to fibular migration than a tether, but the two options provide equivalent clinical outcomes. Consequently, either option may be suitable based on the surgeon’s preference.

## Data Availability

The datasets used and/or analyzed during the current study are available from the corresponding author on reasonable request.
